# Transcutaneous Electrical Nerve Stimulation (TENS) for Opioid-Induced Constipation in Palliative Care: A Systematic Review and Network Meta-Analysis

**DOI:** 10.1155/2023/5383821

**Published:** 2023-04-19

**Authors:** Jianyue Ying, Renzhong Xiao, Lili Xu, Mei Yan

**Affiliations:** ^1^Campus Hospital of Zhejiang University, Zhejiang University, Hangzhou, Zhejiang, China; ^2^Hunan Royal Pharmaceutical Technology Co. Ltd., Changsha, Hunan, China; ^3^School of Pharmacy, Shandong University of Traditional Chinese Medicine, Jinan, Shandong, China; ^4^Jinhua Municipal Central Hospital, Jinhua, Zhejiang, China

## Abstract

**Background:**

Opioid-induced constipation (OIC) has become increasingly prevalent with the rise of prescription opioid use, particularly in patients with advanced illnesses. Existing literature suggests that transcutaneous electrical nerve stimulation (TENS) could be applied to treat cancer pain and reduce OIC incidence. However, there need to be more systematic review studies on the effectiveness of TENS in treating OIC.

**Objective:**

In order to fill the gap of TENS in treating OIC in current knowledge, we have conducted a systematic review and network meta-analysis.

**Methods:**

The comprehensive computer retrieval PubMed, Embase, Cochrane, China National Knowledge Infrastructure (CNKI), Chinese Biomedical (CBM), and Wanfang Database were used to collect literature for relevant studies of TENS treatment of OIC, in accordance with the standard of literature filtering, data extraction, and quality evaluation. The data were meta-analysed using ReviewManager 5.3 software recommended by Cochrane.

**Results:**

A total of 180 pieces of literature were yielded through original search. Based on the inclusion and exclusion criteria, a total of 9 articles were included in this study. Our analysis of seven studies has revealed that TENS (28.18%) significantly reduces the incidence rate of OIC compared to control (52.45%) (*I*^2^ = 57%, *P*=0.03; OR = 0.66 (95% CI, 0.53 to 0.82), *Z* = 3.70, *P* < 0.01). The results of two studies indicated that TENS significantly improved the quality of life compared to the control group (i.e., treatment-as-usual only) (*I*^2^ = 80%, *P*=0.03; OR = −1.91; 95% CI, −2.54 to −1.29, *Z* = 6.00, *P* < 0.01).

**Conclusion:**

The administration of TENS therapy holds the potential to mitigate the occurrence of OIC and augment the quality of life for individuals suffering from cancer. Particularly, TENS therapy proves to be appropriate for propagation within community and domestic environments. Nevertheless, advanced clinical randomized controlled trials of superior quality are necessary to authenticate the comprehensive clinical efficiency and safety of this therapy. Further investigation is indispensable to comprehend its mechanism in greater detail and establish the optimum therapeutic strategy.

## 1. Introduction

Patients with advanced-stage cancer typically experience cancer-associated symptoms, which can considerably impair their quality of life [1]. The prevalence of cancer pain has been estimated to range from 40 to 80%, with its incidence increasing as the disease progresses [2]. It may produce excruciating pain, seriously affecting their quality of life [3]. The World Health Organization (WHO) recommended opioids as analgesics to treat cancer pain, and they are widely used clinically for managing chronic cancer pain [4]. However, previous studies have suggested that opioid-induced constipation (OIC) can occur in up to 90% of patients, which is often persistent [5, 6]. Therefore, minimizing cancer pain and its side effects is essential to manage cancer pain. To treat OIC, pharmacological and nonpharmacological treatment approaches have been studied. Although the clinical use of oral laxatives or rectal administration can relieve constipation, their adverse reactions are noticeable, and their effectiveness is limited. Transcutaneous electrical nerve stimulation (TENS) is a safe, noninvasive, relatively inexpensive, and nonpharmacological approach as an adjunctive analgesic treatment for advanced cancer pain [7–9]. TENS involves a cutaneous electrode that is equivalent to needleless electroacupuncture. It can stimulate the release of endogenous neurotransmitters such as endorphin and dynorphin and enhance the maintenance of normal physiological movement of the gastrointestinal tract [10]. Several studies have suggested that TENS can significantly improve the clinical symptoms and quality of life in patients with OIC [11–13]. However, Nakano et al. [14] posit that while TENS can significantly reduce opioid side effects such as nausea, pain, and OIC, its effects on OIC treatment are not satisfactory.

Currently, a limited amount of literature is available on the use of TENS for treating OIC, and a systematic review of the research literature was not found. We conducted a systematic review and network meta-analysis to address this knowledge gap.

## 2. Methods

### 2.1. Search Strategy

We systematically searched several databases, including PubMed, Embase, Cochrane, China National Knowledge Infrastructure (CNKI), Chinese Biomedical (CBM), and Wanfang Database, from inception to July 1, 2022, without language restrictions. The search terms included “cancer,” “neoplasm,” “tumor,” “constipation,” and “Transcutaneous Electric Nerve Stimulation.” The keywords were combined using Boolean searches and MeSH descriptors. The detailed search strategy is shown in Table 1. The search strategy was formulated by two independent researchers who conducted a pre-examination, checked, and finalized the strategies before conducting a formal search. In the event of any disagreement, both researchers discussed and reached a consensus, and if necessary, a third party was consulted for a final decision.

### 2.2. Study Selection Criteria

The inclusion criteria for the study were as follows: (a) randomized controlled trials or other controlled studies; (b) cancer patients diagnosed with OIC, regardless of race, sex, age, disease course, and severity; (c) description of the opioid dosage; (d) the treatment group was treated with TENS, while patients in the control group received rehabilitation treatment but did not receive TENS therapy; (e) the results reported the incidence of constipation or constipation scores; (f) double-blind and open-label trial designs were eligible. The exclusion criteria were: (a) non-English and non-Chinese language articles, abstracts from conferences, full texts without raw data for retrieval, duplicate publications, letters, and review articles; and (b) articles that did not meet the inclusion criteria.

### 2.3. Data Extraction and Quality Assessment

Two reviewers independently extracted data from the studies and assessed their eligibility for inclusion based on the abovementioned criteria. Any discrepancies between the two reviewers were resolved through consensus or discussion with a third reviewer. The extracted data included: (a) basic study information, such as first author and publication year; (b) study characteristics, including sample size, patient age, analgesic measures, types of cancer, treatment time, TENS-related information (electrical stimulation points, electrical nerve stimulator parameter setting, frequency); (c) primary outcome measures of interest, including the incidence of constipation or the effective rate of treating constipation, secondary outcome measures, such as quality of life scores, and the incidence of adverse reactions; and (d) relevant elements of the bias risk assessment.

Conducting randomized clinical trials of interventions such as TENS or acupuncture present challenges in achieving blinding, which refers to the extent to which participants and/or researchers must be made aware of which intervention is being administered. Blinding is crucial as it can minimize bias due to the expectations or preferences of participants and researchers. However, blinding may be difficult or impossible in some interventions, such as physical manipulation or sensory stimulation. Although such trials may not be blinded, they are still considered acceptable by researchers [15–18]. The Cochrane collaboration tool is commonly used to assess selection bias in meta-analyses of randomized controlled clinical trials [19]. It evaluates whether data allocation was random and whether any baseline differences between groups suggest issues with randomization [20]. This is precisely the objective of our study, and thus, we chose the Cochrane collaboration tool to evaluate the risk of bias. Two review authors evaluated the methodological quality of included trials and their risk of bias across seven domains, including allocation concealment, random sequence generation, incomplete outcome data, selective outcome reporting, blinding of participants and personnel, blinding of outcome assessment, and other biases, based on the Cochrane collaboration's tool [21].

### 2.4. Statistical Analysis

We used RevMan 5.3 (Cochrane Collaboration) software to conduct the meta-analyses. For dichotomous variables, we used the odds ratio (OR) and 95% confidence interval (95% CI). The choice between the fixed effect and random effect model in meta-analysis depended on the degree of heterogeneity among the studies, as determined by the previous literature [22, 23]. Specifically, we used the fixed effect model for meta-analysis if *P* > 0.10 and *I*^2^ ≤ 50% and the random effect model if *P* ≤ 0.10 and *I*^2^ > 50%. In cases where clinical heterogeneity was too significant or insufficient literature to perform a meta-analysis, we used descriptive analysis. We considered *P* < 0.05 to be a significant difference.

## 3. Results

### 3.1. Results Included Studies

**Figure 1:**
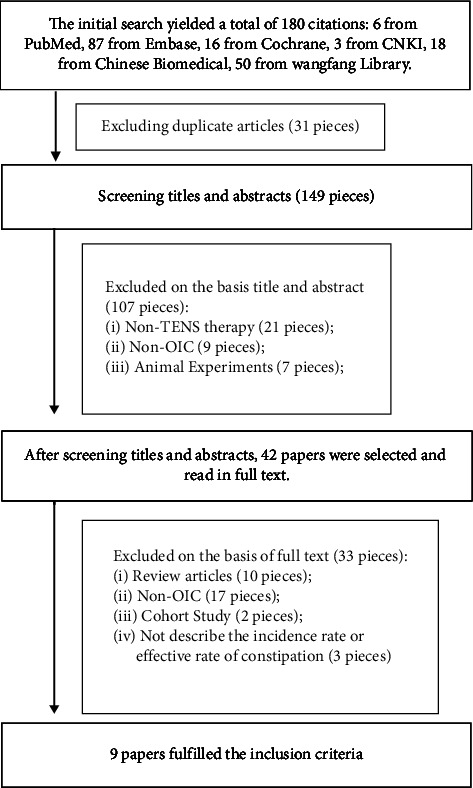
Literature selection process and results.

**Figure 2:**
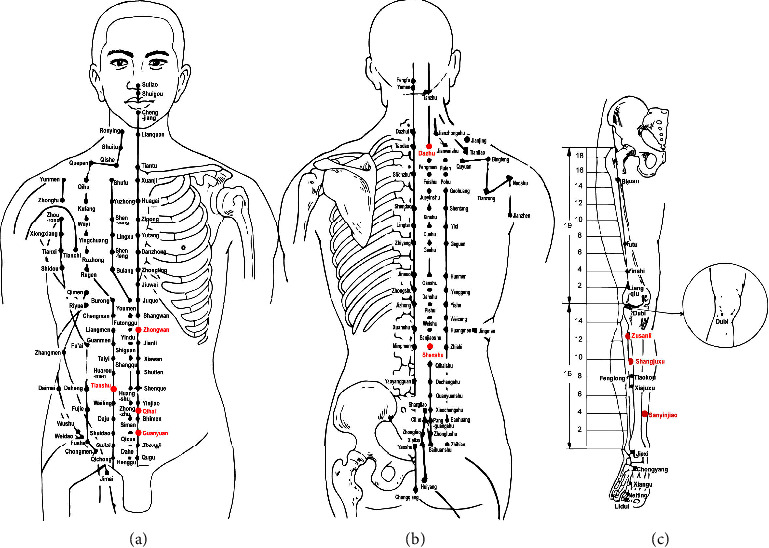
Schematic diagram of the acupoints in the included articles. Notes: Dazhu (BL 11): in the back, under the spinous process of the first thoracic vertebra, beside 5 cm; Shenshu (BL 23): both sides of the spinous process of a second lumbar vertebra; Zusanli (ST 36): 3-4 mm below the knee joint and 1-2 mm outside the tibia crest; Sanyinjiao (SP 6): 10 mm proximal to the prominence of the medial malleolus; Guanyuan (RN 4): 3 cm below the belly button; Qihai (RN 6): 1.5 cm below the belly button; Tianshu (ST 25): 2 cm to the side of the belly button; Shangjuxu (ST 37): 7-8 mm below the knee joint and 1-2 mm outside the tibia crest; Zhongwan (RN 12): 4 cm above the belly button.

In our initial search, we identified 180 pieces of literature, out of which 31 were excluded due to duplication, resulting in a total of 149 studies for title and abstract screening. After this screening, 42 articles were selected for full-text reading, out of which 33 were excluded as they did not meet the eligibility criteria. Finally, a total of 9 studies that met the standards were included in the meta-analysis. A summary of the literature search process is presented in Figure 1. The primary characteristics of the included articles are shown in Table 2, and the acupoints utilized in the included studies are shown in Figure 2.

### 3.2. Risk of Bias Assessment

**Figure 3:**
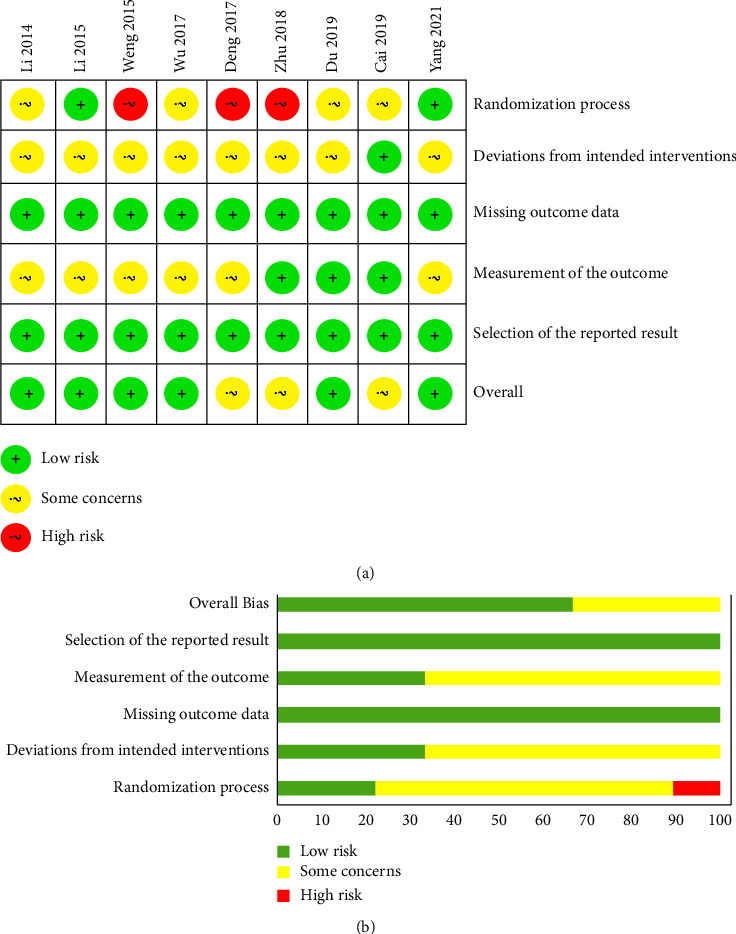
Quality assessment of included studies; the green circles indicate lack of bias; yellow circles indicate unclear bias. (a) Risk of bias for each included study. (b) The overall summary of bias of the included studies.

We assessed the risk of bias and concerns regarding the applicability of the included studies using the Cochrane collaboration tool (ROB 2 IRPG 2018 beta v6), and the results are reported in Figure 3. Overall, the included studies were of high quality, with only some concerns regarding allocation concealment, study subjects hiding, and researcher hiding being rated as a significant risk of bias. However, it is generally acceptable for these factors to be rated as high risk of bias because blinded studies involving TENS or acupuncture can be challenging to conduct [15–18].

### 3.3. Primary Outcomes: Incidence Rate of OIC or Effective Rate

**Figure 4:**
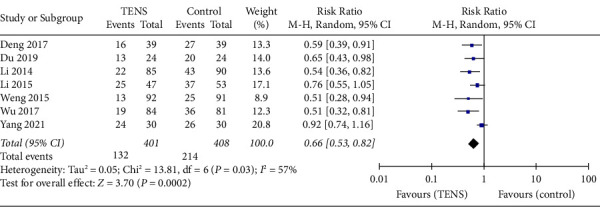
Forest plot of the meta-analysis of the incidence rate of OIC between TENS and control groups.

A total of seven studies (809 participants; TENS: 401, control: 408) that reported the incidence rate of OIC provided sufficient data for statistical pooling. The test for the heterogeneity among the studies showed significant heterogeneity (*P*=0.03; *I*^2^ = 57%), and as such, the random-effects model was used. The meta-analysis results are presented in Figure 4. The sample size was 809, consisting of 401 cases (OIC occurred in 113 patients) and 408 controls (OIC occurred in 214 patients). The heterogeneity, calculated using the *I*^2^ statistic with a random-effect model, was *I*^2^ = 57%, *P*=0.03. Overall, TENS significantly reduced the incidence rate of OIC compared with the control (pooled OR = 0.66; 95% CI: 0.53–0.82, *Z* = 3.70, *P*  <  0.01).

**Figure 5:**
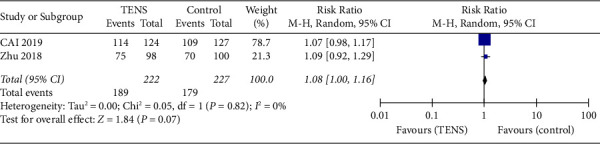
Forest plot of the meta-analysis of the effective rate of treating OIC between TENS and control groups.

Two studies (449 participants; TENS: 222, control: 227) compared the effective rate of treating constipation between the TENS treatment group and the control group. The test for the heterogeneity among the studies showed significant heterogeneity (*P*=0.82; *I*^2^ = 0%), and as such, the random-effects model was used. The meta-analysis results showed in Figure 5. The meta-analysis results showed no statistically significant effect of TENS in increasing the effective rate of treating OIC compared with control (lactulose oral solution) (*I*^2^ = 0%; *P*=0.82; OR = 1.08; 95% CI: 1.00–1.16, *Z* = 1.84, *P*=0.07).

### 3.4. Secondary Outcomes: Quality of Life Scores

**Figure 6:**
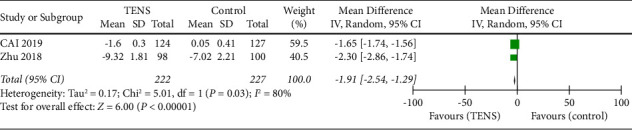
Forest plot of the meta-analysis of quality of life scores between TENS and control groups.

Two studies (449 participants; TENS: 222, control: 227) compared the quality of life scores between the TENS treatment group and the control group. The test for the heterogeneity among the studies showed significant heterogeneity (*P*=0.03; *I*^2^ = 80%), and as such, the random-effects model was used. The meta-analysis results are presented in Figure 6. Overall, TENS significantly increased the quality of life compared with control (*I*^2^ = 80%, *P*=0.03; OR = −1.91; 95% CI: −2.54 to −1.29, *Z* = 6.00, *P* < 0.01).

## 4. Discussion

TENS was employed to ameliorate the adverse effects of chemotherapy, such as nausea, insomnia, cachexia, and pain, in patients with advanced cancer. Although it cannot be considered a substitute for opioids and other pharmacological interventions, it can support palliative care [12]. Compared to the traditional acupuncture point treatment, TENS is uncomplicated, safe, reproducible, and more appropriate for long-term home patients who cannot take oral medications for self-management, deserving of clinical promotion. The efficacy of TENS in cancer patients is not only limited to pain relief but also extends to mitigating other physical symptoms during palliative care, including constipation, fatigue, nausea, and vomiting, as reported in various studies [13, 32, 33].

Reportedly, 97% of patients with cancer and OIC experience moderate to severe constipation-related symptoms, despite the widespread application of laxatives [34]. However, the present meta-analysis findings unveil that the incidence of OIC is 66%, and TENS markedly curtails the incidence rate of OIC compared to the control group. The outcomes of this study should push for further research to compare the incidence rate of OIC and bowel function index across various types of cancer.

The precise selection of points is paramount for the efficient employment of TENS in alleviating OIC or cancer-related discomfort. According to the comprehensive review of nine OIC prevention studies, the Tianshu acupoint was identified as the optimal point for selection, and a meta-analysis confirmed its efficacy in significantly reducing the incidence of OIC.

Point selection is essential for TENS to treat OIC or cancer pain. The nine OIC prevention studies included in this review found that Tianshu acupoint was selected, and a meta-analysis showed that it could significantly reduce the incidence of OIC. There are few studies on TENS therapy or adjuvant therapy for OIC, and only 9 studies were searched in this study. The dearth of knowledge on the specific mechanisms of OIC, coupled with the numerous OIC cases, necessitates more in-depth and meticulous research in the future, such as stimulating different acupoints for the effect of comparative studies, different frequency stimulation effects of comparative studies, different types of cancer, and psychological interventions. Ideally, future studies should be randomized, double-blinded, and placebo-controlled to assess TENS' clinical efficacy and safety. However, this is an enormous challenge, as the blindness of TEAS treatment seems impossible, given that patients will eventually know whether they receive treatment. Nakano et al. [12] studied the constipation of patients in TENS and non-TENS phases, but whether there is a difference in the extent of the constipation of cancer is controversial. We recommend random TENS treatment, that is, a random selection of patients after admission for five days of TENS or five days of lactulose oral liquid treatment, which may be more adept at reducing the error.

TENS may possess a mechanism of action akin to that of acupuncture. The mechanism of pain treatment via acupuncture centers around the activation of opioid receptors and neuropeptide genes [35]. Acupuncture regulates brain-gut peptide metabolism by controlling the functional activity of the central nervous system, which in turn regulates the brain-gut axis, possibly being the mechanism of acupuncture. Furthermore, a crucial element of the acupuncture effect is regulating and repairing the nervous system, regulating emotions, and mitigating patients' emotions [36–39]. Research needs to be done more regarding the mechanism of TENS or acupuncture for treating OIC.

Chemotherapy patients often endure the pain caused by chemotherapy-induced adverse reactions, and due to the nature of their treatment, they commonly reside in their homes during the chemotherapy interval. Consequently, nurses are faced with the challenge of monitoring their condition and providing adequate care. The evolution of family rehabilitation and community health care as a means of continuing care has been slow, and nursing personnel generally lack a comprehension of the concept of continuing care. Given the remedial effects of acupuncture on analgesia, there has been a growing acceptance of acupuncture treatment. TENS, a new type of acupuncture, has a similar analgesic effect to acupuncture [40] but with the advantage of being noninvasive, thus making it more readily accepted [41, 42]. Providing appropriate physical treatment services, including early intervention and community follow-up, can significantly aid in maintaining the functional independence and quality of life of patients receiving palliative treatment [43].

Several limitations were present in this meta-analysis. Firstly, it is noteworthy that meta-analysis represents secondary research, and the outcome significantly relies on primary studies [21], inevitably leading to certain limitations. Secondly, sample sizes in some of the included studies and subgroup analyses were comparatively small. Consequently, the limited sample sizes might bias the outcome. Thirdly, researchers find it challenging to guarantee strict consistency when they conduct research under the control of related factors criteria, such as age range, physical condition, types of cancer, and cancer stages of the included populations, which will affect the research outcome some degree. Fourthly, the publication bias test displayed no evidence of publication bias.

Nevertheless, this research conducted a comprehensive retrieval with a language limited to Chinese and English. Hence, it may lead to corresponding language bias, even though it is understood that accessibility is limited to databases and language ability. Fifthly, the lack of standardization is an issue. Currently, there is no standardized protocol for TENS treatment for OIC. The optimal frequency, intensity, and duration of treatment vary depending on the individual's response. Most studies on TENS have focused on treating pain rather than constipation. In conclusion, we must take the findings of this study into account.

## 5. Conclusion

With the progress of technology, it is possible to individualize TENS therapy based on an individual's response. Especially, TENS is suitable for promotion in the community and home settings. In conclusion, TENS can potentially treat OIC, but the evidence of its efficacy is limited. Further research is essential to comprehend its mechanism better and establish the optimal therapeutic approach. TENS can be combined with other therapies to provide complete relief for OIC.

## Figures and Tables

**Table 1:** The detailed searching strategy of our systematic review.

Data source	Search terms
PubMed	((((cancer [MeSH Terms]) OR (neoplasm [MeSH Terms])) OR (tumor [MeSH Terms])) AND (constipation [MeSH Terms])) AND (electrical nerve stimulation)
Embase	(“cancer” OR “neoplasm” OR “tumor”) AND “constipation”/exp AND electrical AND nerve AND stimulation
Cochrane	(MeSH descriptor: [neoplasms] explode all trees) AND (MeSH descriptor: [Opioid-Induced Constipation] explode all trees) AND (MeSH descriptor: [Transcutaneous Electric Nerve Stimulation] explode all trees)
CNKI	Topic:(neoplasms) AND Topic:(Constipation) AND Topic:(electrical nerve stimulation)
CBM	neoplasms [intelligence] AND constipation [intelligence] AND electrical nerve stimulation [intelligence]
Wanfang Database	Topic:(neoplasms) AND Topic:(Constipation) AND Topic:(electrical nerve stimulation)

**Table 2:** Summaries of the interventions and clinical outcomes in this study.

Study, year (reference)	Age		Participants, *n*		Analgesic measures		Types of cancer	Interventions		Electrical stimulation points	Treatment protocols for TENS	Constipation	
	Treatment group	Control group	Treatment group	Control group	Treatment group	Control group		Treatment group	Control group			Treatment group	Control group
Li, 2014 [24]	52.9 ± 18.3	53.2 ± 18.5	85	90	OHDT and TENS	OHDT: gradually adjusted until a stable analgesic dose	Multiple types of cancer	TENS	N/A	Tianshu	N/A	IR: 22/85 (25.9%)	IR: 43/90 (47.8%)

Li, 2015 [25]	N/A	N/A	47	53	OHDT and TENS	OHDT: gradually adjusted until a stable analgesic dose	Multiple types of cancer	TENS	N/A	Tianshu	N/A	IR: 25/47 (53.2%)	IR: 37/53 (69.8%)

Weng, 2015 [26]	62.5 ± 11.3	65.1 ± 10.8	92	91	OHDT and TENS	OHDT: gradually adjusted until a stable analgesic dose	Multiple types of cancer	TENS	N/A	Tianshu	N/A	IR: 13/92 (14.13%)	IR: 25/91 (27.47%)

Wu, 2017 [27]	47.41 ± 11.84	49.13 ± 10.38	84	81	OHDT, TENS, and oral Xiaoji Zhitong decoction	OHDT: gradually adjusted until a stable analgesic dose	Multiple types of cancer	TENS	N/A	Tianshu	N/A	IR: 19/84 (10.7%)	IR: 36/81 (44.4%)

Deng, 2017 [28]	56.1.1 ± 4.6		39	39	OHDT and TENS	OHDT: gradually adjusted until a stable analgesic dose	Multiple types of cancer	TENS	N/A	Tianshu	30 consecutive days, the stimulation current intensity gradually increased so that the patient could still tolerate it	IR: 16/39 (41.0%)	IR: 27/39 (69.2%)

Du, 2019 [29]	59.1 ± 1.6	58.2 ± 1.6	24	24	OHDT and TENS	OHDT: gradually adjusted until a stable analgesic dose	Bone metastases cancer	TENS	N/A	Dazhu; Shenshu; Zusanli; Sanyinjiao	30 min/time, 1 time/d, 4 consecutive weeks; electrical nerve stimulator parameter setting: a dilatational wave (frequency 2–100 Hz) and bearable current intensity were used	IR: 13/24 (54.2%)	IR: 20/24 (83.3%)
Yang, 2021 [30]	63.87 ± 9.02	64.43 ± 10.92	30	30	OHDT and TENS	OHDT: WHO pain treatment paradigm	Multiple types of cancer	TENS	N/A	Kongzui; Taichong; Siguan; Zusanli; Neiguan	20−30 min/time, 1 time/d, 7 consecutive days; electrical nerve stimulator parameter setting: a dilatational wave (frequency 2/100 Hz) and bearable current intensity were used	IR: 24/30 (80.00%)	IR: 26/30 (86.67%)

Zhu, 2018 [31]	57.12 ± 9.93	59.54 ± 10.77	58	60	OHDT and TENS	OHDT: ≥80 mg/day	No description	TENS	Lactulose oral solution	Tianshu; Zhongwan	80–120 Hz; 30 min/day, 2 consecutive weeks	ER: (76.5%)	70.00%
												PAC-QOL: −9.32 ± 1.81	−7.02 ± 2.21

Cai, 2019 [11]	51.2 ± 3.4	55.3 ± 3.1	124	127	OHDT and TENS	OHDT: gradually adjusted until a stable analgesic dose	Multiple types of cancer	TENS	Lactulose oral solution	Guanyuan; Qihai; Tianshu; Zusanli; Shangjuxu	30 min/time, 1 time/d, 14 consecutive days; electrical nerve stimulator parameter setting: a dilatational wave (frequency 2/100 Hz) and bearable current intensity were used	ER: 114/124 (91.9%)	ER: 109/127 (85.8%)
												PAC-QOL: −1.6 ± 0.3	0.05 ± 0.41

OHDT, oxycodone hydrochloride delayed-release tablets; TENS, transcutaneous electrical nerve stimulation; IR, incidence rate; ER, effective rate; NCCN clinical practice guidelines in oncology (version 2 2011) and the following formulas: transdermal fentanyl (25 mcg/h) ≈ oral oxycodone (30 mg/d) ≈ parenteral morphine (20 mg/day) ≈ oral morphine (60 mg/day).
